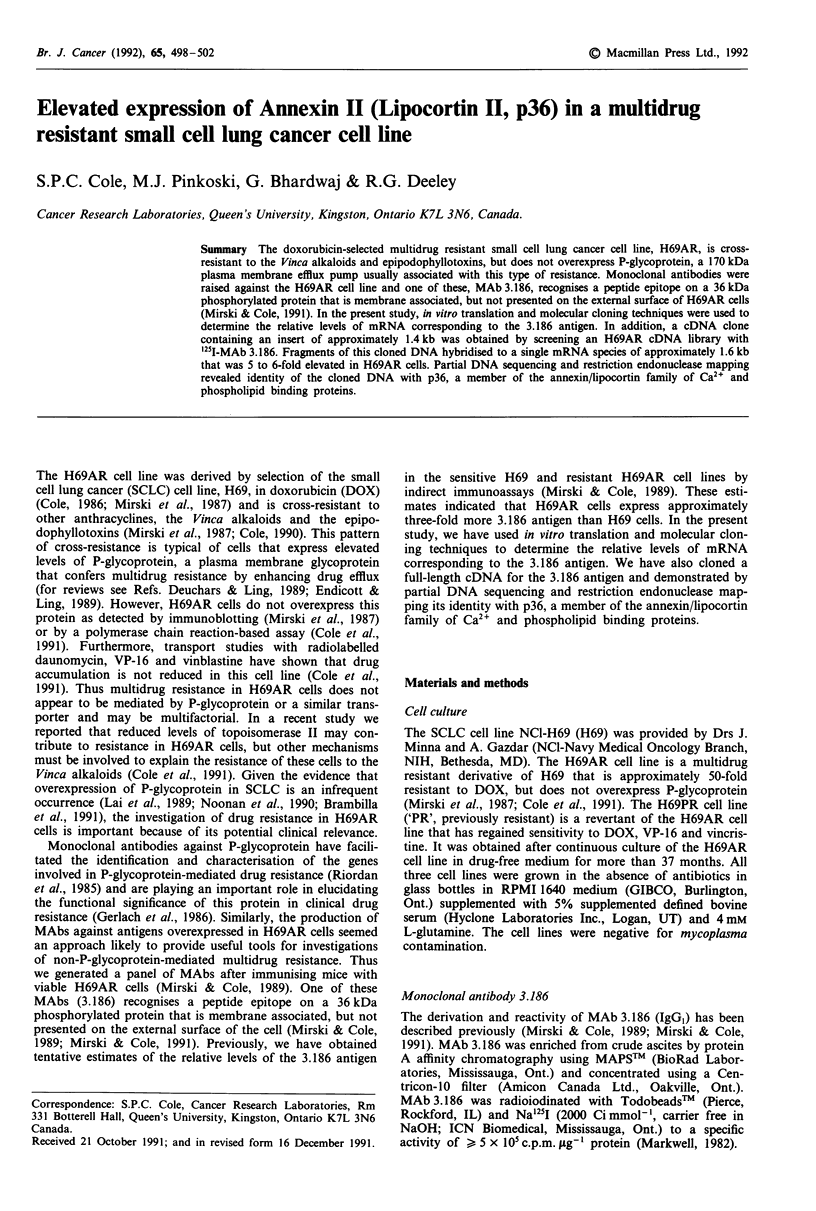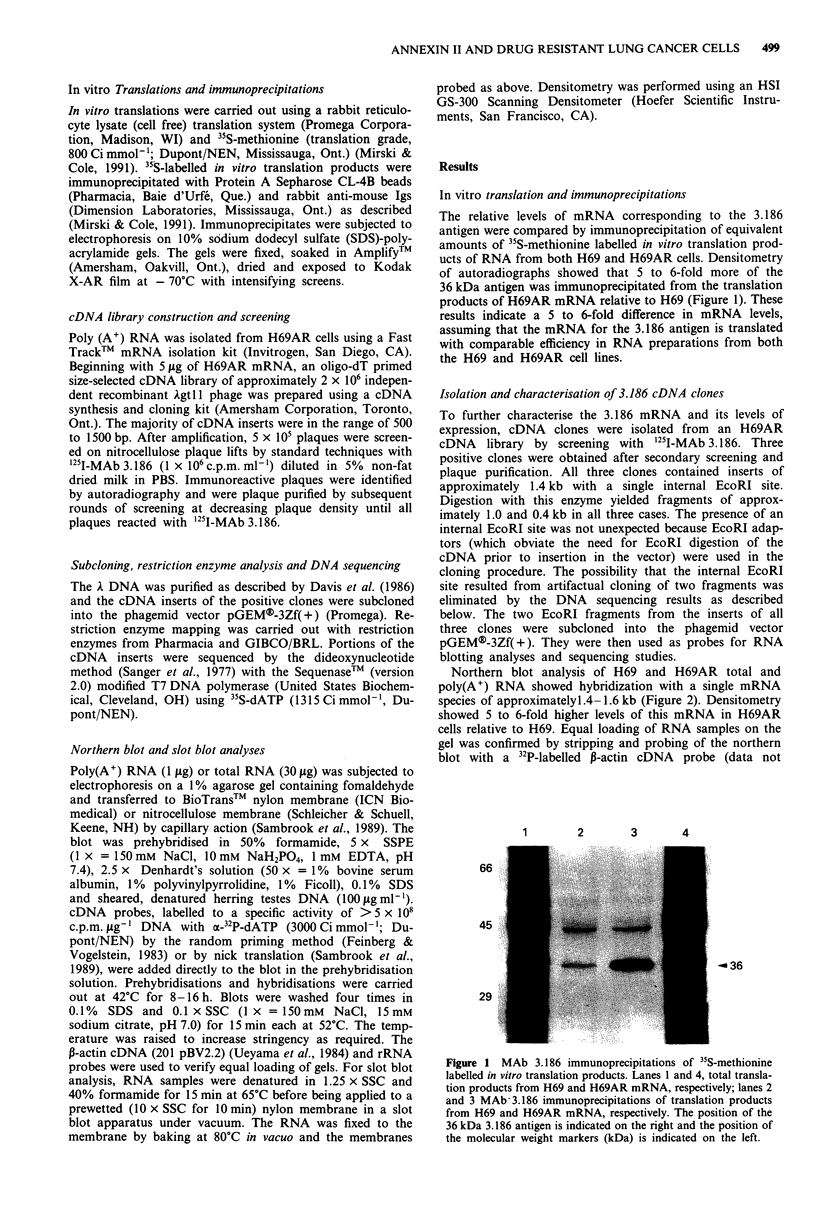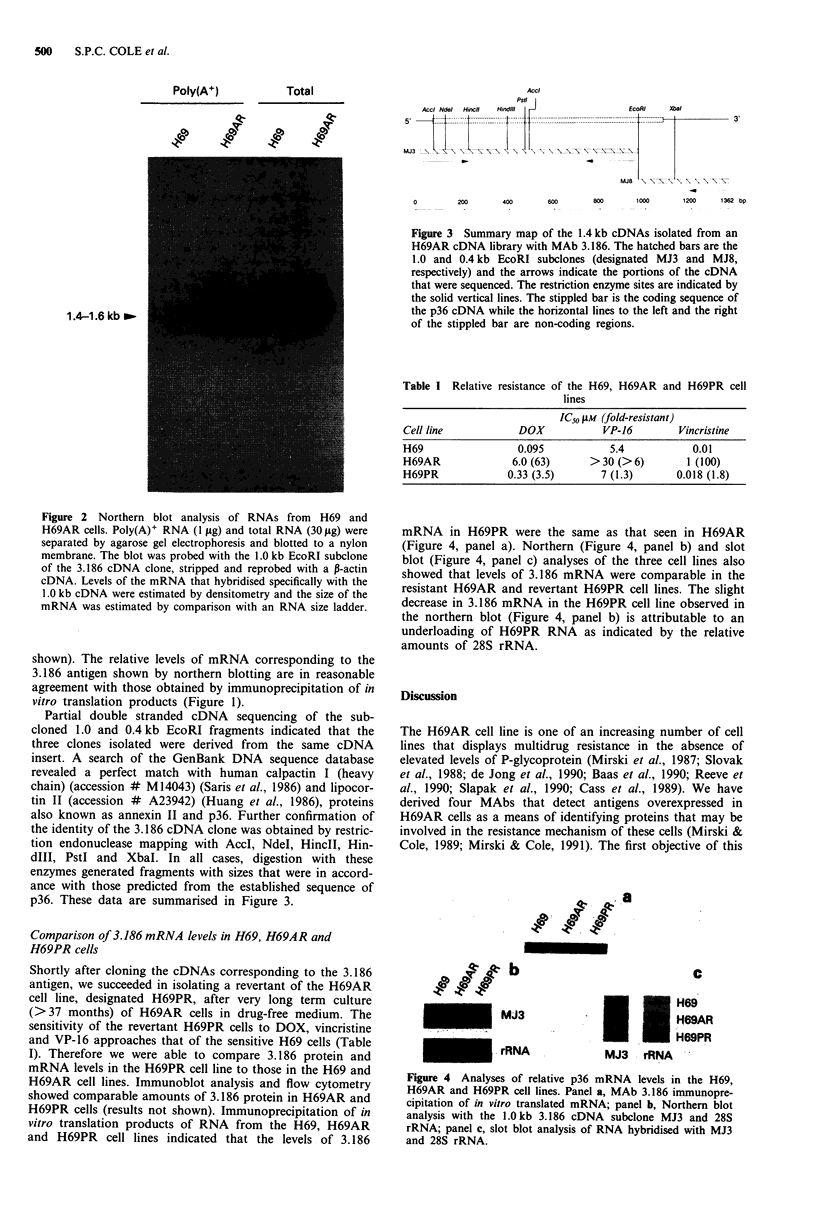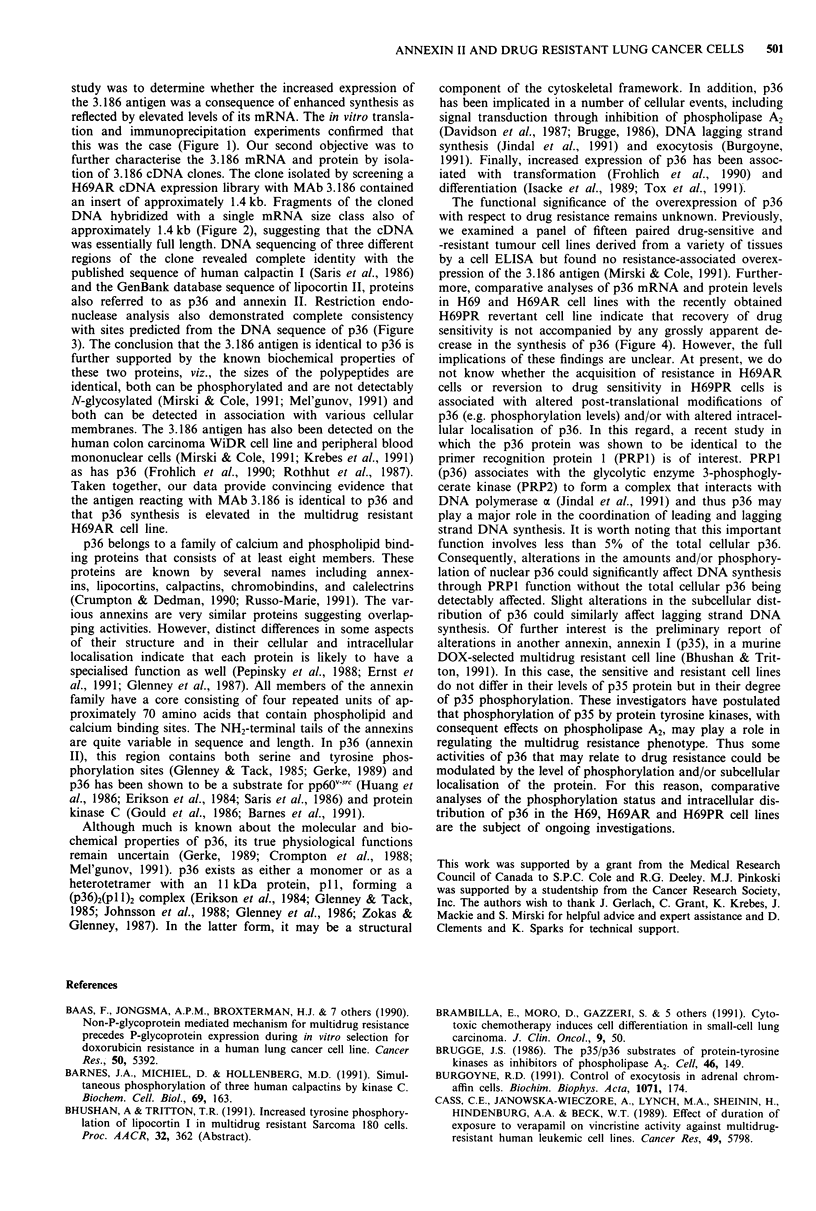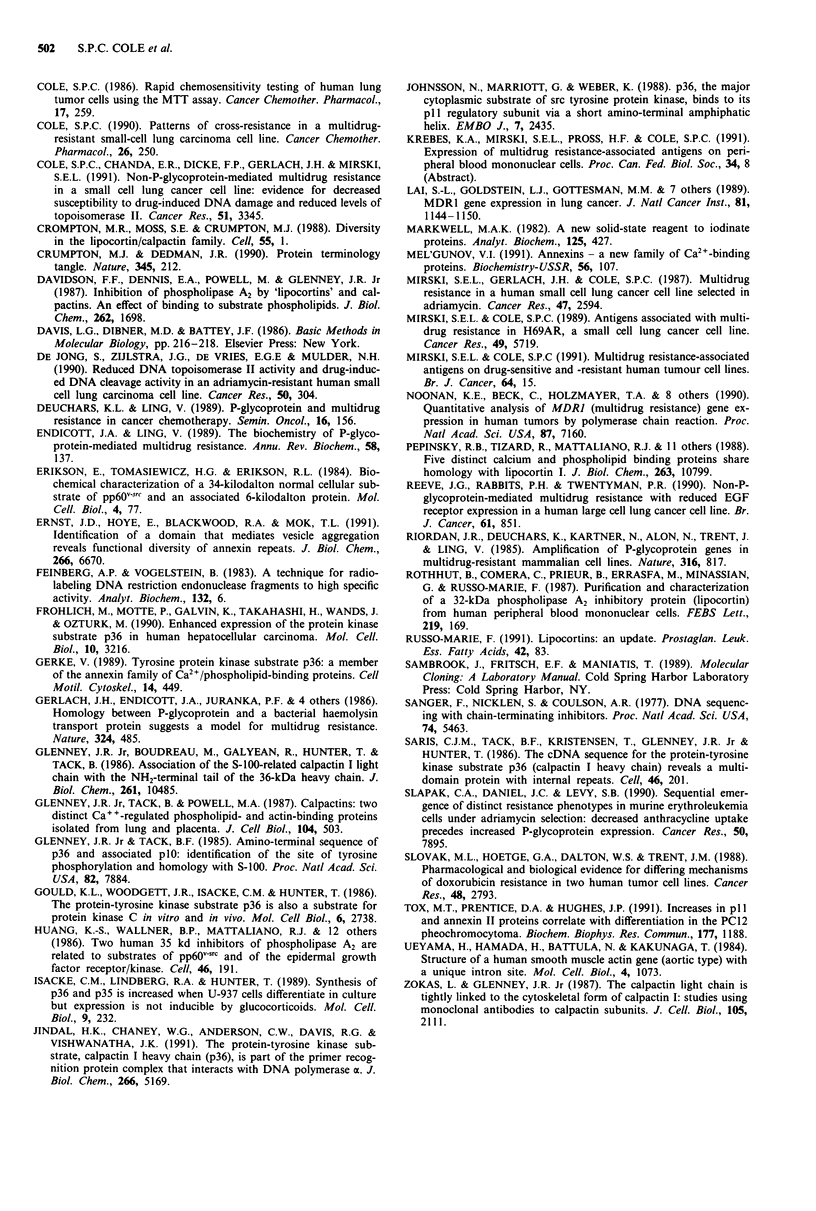# Elevated expression of annexin II (lipocortin II, p36) in a multidrug resistant small cell lung cancer cell line.

**DOI:** 10.1038/bjc.1992.103

**Published:** 1992-04

**Authors:** S. P. Cole, M. J. Pinkoski, G. Bhardwaj, R. G. Deeley

**Affiliations:** Cancer Research Laboratories, Queen's University, Kingston, Ontario, Canada.

## Abstract

**Images:**


*Br. J. Cancer* (1992), **65**, 498–502 © Macmillan Press Ltd., 1992

### Elevated expression of Annexin II (Lipocortin II, p36) in a multidrug resistant small cell lung cancer cell line

S.P.C. Cole, M.J. Pinkoski, G. Bhardwaj & R.G. Deeley

*Cancer Research Laboratories, Queen's University, Kingston, Ontario K7L 3N6, Canada.*

**Summary** The doxorubicin-selected multidrug resistant small cell lung cancer cell line, H69AR, is cross-resistant to the *Vinca* alkaloids and epipodophyllotoxins, but does not overexpress P-glycoprotein, a 170 kDa plasma membrane efflux pump usually associated with this type of resistance. Monoclonal antibodies were raised against the H69AR cell line and one of these, MAb 3.186, recognises a peptide epitope on a 36 kDa phosphorylated protein that is membrane associated, but not presented on the external surface of H69AR cells (Mirski & Cole, 1991). In the present study, *in vitro* translation and molecular cloning techniques were used to determine the relative levels of mRNA corresponding to the 3.186 antigen. In addition, a cDNA clone containing an insert of approximately 1.4 kb was obtained by screening an H69AR cDNA library with $^{125}$I-MAb 3.186. Fragments of this cloned DNA hybridised to a single mRNA species of approximately 1.6 kb that was 5 to 6-fold elevated in H69AR cells. Partial DNA sequencing and restriction endonuclease mapping revealed identity of the cloned DNA with p36, a member of the annexin/lipocortin family of $Ca^{2+}$ and phospholipid binding proteins.

The H69AR cell line was derived by selection of the small cell lung cancer (SCLC) cell line, H69, in doxorubicin (DOX) (Cole, 1986; Mirski *et al.*, 1987) and is cross-resistant to other anthracyclines, the *Vinca* alkaloids and the epipodophyllotoxins (Mirski *et al.*, 1987; Cole, 1990). This pattern of cross-resistance is typical of cells that express elevated levels of P-glycoprotein, a plasma membrane glycoprotein that confers multidrug resistance by enhancing drug efflux (for reviews see Refs. Deuchars & Ling, 1989; Endicott & Ling, 1989). However, H69AR cells do not overexpress this protein as detected by immunoblotting (Mirski *et al.*, 1987) or by a polymerase chain reaction-based assay (Cole *et al.*, 1991). Furthermore, transport studies with radiolabelled daunomycin, VP-16 and vinblastine have shown that drug accumulation is not reduced in this cell line (Cole *et al.*, 1991). Thus multidrug resistance in H69AR cells does not appear to be mediated by P-glycoprotein or a similar transporter and may be multifactorial. In a recent study we reported that reduced levels of topoisomerase II may contribute to resistance in H69AR cells, but other mechanisms must be involved to explain the resistance of these cells to the *Vinca* alkaloids (Cole *et al.*, 1991). Given the evidence that overexpression of P-glycoprotein in SCLC is an infrequent occurrence (Lai *et al.*, 1989; Noonan *et al.*, 1990; Brambilla *et al.*, 1991), the investigation of drug resistance in H69AR cells is important because of its potential clinical relevance.

Monoclonal antibodies against P-glycoprotein have facilitated the identification and characterisation of the genes involved in P-glycoprotein-mediated drug resistance (Riordan *et al.*, 1985) and are playing an important role in elucidating the functional significance of this protein in clinical drug resistance (Gerlach *et al.*, 1986). Similarly, the production of MAbs against antigens overexpressed in H69AR cells seemed an approach likely to provide useful tools for investigations of non-P-glycoprotein-mediated multidrug resistance. Thus we generated a panel of MAbs after immunising mice with viable H69AR cells (Mirski & Cole, 1989). One of these MAbs (3.186) recognises a peptide epitope on a 36 kDa phosphorylated protein that is membrane associated, but not presented on the external surface of the cell (Mirski & Cole, 1989; Mirski & Cole, 1991). Previously, we have obtained tentative estimates of the relative levels of the 3.186 antigen in the sensitive H69 and resistant H69AR cell lines by indirect immunoassays (Mirski & Cole, 1989). These estimates indicated that H69AR cells express approximately three-fold more 3.186 antigen than H69 cells. In the present study, we have used *in vitro* translation and molecular cloning techniques to determine the relative levels of mRNA corresponding to the 3.186 antigen. We have also cloned a full-length cDNA for the 3.186 antigen and demonstrated by partial DNA sequencing and restriction endonuclease mapping its identity with p36, a member of the annexin/lipocortin family of $Ca^{2+}$ and phospholipid binding proteins.

Correspondence: S.P.C. Cole, Cancer Research Laboratories, Rm 331 Botterell Hall, Queen's University, Kingston, Ontario K7L 3N6 Canada.
Received 21 October 1991; and in revised form 16 December 1991.

### Materials and methods

### *Cell culture*

The SCLC cell line NCl-H69 (H69) was provided by Drs J. Minna and A. Gazdar (NCl-Navy Medical Oncology Branch, NIH, Bethesda, MD). The H69AR cell line is a multidrug resistant derivative of H69 that is approximately 50-fold resistant to DOX, but does not overexpress P-glycoprotein (Mirski *et al.*, 1987; Cole *et al.*, 1991). The H69PR cell line ('PR', previously resistant) is a revertant of the H69AR cell line that has regained sensitivity to DOX, VP-16 and vincristine. It was obtained after continuous culture of the H69AR cell line in drug-free medium for more than 37 months. All three cell lines were grown in the absence of antibiotics in glass bottles in RPMI 1640 medium (GIBCO, Burlington, Ont.) supplemented with 5% supplemented defined bovine serum (Hyclone Laboratories Inc., Logan, UT) and 4 mM L-glutamine. The cell lines were negative for *mycoplasma* contamination.

### *Monoclonal antibody 3.186*

The derivation and reactivity of MAb 3.186 ($IgG_1$) has been described previously (Mirski & Cole, 1989; Mirski & Cole, 1991). MAb 3.186 was enriched from crude ascites by protein A affinity chromatography using MAPS™ (BioRad Laboratories, Mississauga, Ont.) and concentrated using a Centricon-10 filter (Amicon Canada Ltd., Oakville, Ont.). MAb 3.186 was radioiodinated with Todobeads™ (Pierce, Rockford, IL) and $Na^{125}I$ (2000 Ci $mmol^{-1}$, carrier free in NaOH; ICN Biomedical, Mississauga, Ont.) to a specific activity of $\geqslant 5 \times 10^5$ c.p.m. $\mu g^{-1}$ protein (Markwell, 1982).

### In vitro *Translations and immunoprecipitations*

*In vitro* translations were carried out using a rabbit reticulocyte lysate (cell free) translation system (Promega Corporation, Madison, WI) and $^{35}$S-methionine (translation grade, 800 Ci mmol$^{-1}$; Dupont/NEN, Mississauga, Ont.) (Mirski & Cole, 1991). $^{35}$S-labelled *in vitro* translation products were immunoprecipitated with Protein A Sepharose CL-4B beads (Pharmacia, Baie d'Urfé, Que.) and rabbit anti-mouse Igs (Dimension Laboratories, Mississauga, Ont.) as described (Mirski & Cole, 1991). Immunoprecipitates were subjected to electrophoresis on 10% sodium dodecyl sulfate (SDS)-polyacrylamide gels. The gels were fixed, soaked in Amplify™ (Amersham, Oakvill, Ont.), dried and exposed to Kodak X-AR film at − 70°C with intensifying screens.

### *cDNA library construction and screening*

Poly (A$^+$) RNA was isolated from H69AR cells using a Fast Track™ mRNA isolation kit (Invitrogen, San Diego, CA). Beginning with 5 µg of H69AR mRNA, an oligo-dT primed size-selected cDNA library of approximately 2 × 10$^6$ independent recombinant λgt11 phage was prepared using a cDNA synthesis and cloning kit (Amersham Corporation, Toronto, Ont.). The majority of cDNA inserts were in the range of 500 to 1500 bp. After amplification, 5 × 10$^5$ plaques were screened on nitrocellulose plaque lifts by standard techniques with $^{125}$I-MAb 3.186 (1 × 10$^6$ c.p.m. ml$^{-1}$) diluted in 5% non-fat dried milk in PBS. Immunoreactive plaques were identified by autoradiography and were plaque purified by subsequent rounds of screening at decreasing plaque density until all plaques reacted with $^{125}$I-MAb 3.186.

### *Subcloning, restriction enzyme analysis and DNA sequencing*

The λ DNA was purified as described by Davis *et al.* (1986) and the cDNA inserts of the positive clones were subcloned into the phagemid vector pGEM®-3Zf(+) (Promega). Restriction enzyme mapping was carried out with restriction enzymes from Pharmacia and GIBCO/BRL. Portions of the cDNA inserts were sequenced by the dideoxynucleotide method (Sanger *et al.*, 1977) with the Sequenase™ (version 2.0) modified T7 DNA polymerase (United States Biochemical, Cleveland, OH) using $^{35}$S-dATP (1315 Ci mmol$^{-1}$, Dupont/NEN).

### *Northern blot and slot blot analyses*

Poly(A$^+$) RNA (1 µg) or total RNA (30 µg) was subjected to electrophoresis on a 1% agarose gel containing fomaldehyde and transferred to BioTrans™ nylon membrane (ICN Biomedical) or nitrocellulose membrane (Schleicher & Schuell, Keene, NH) by capillary action (Sambrook *et al.*, 1989). The blot was prehybridised in 50% formamide, 5 × SSPE (1 × = 150 mM NaCl, 10 mM $NaH_2PO_4$, 1 mM EDTA, pH 7.4), 2.5 × Denhardt's solution (50 × = 1% bovine serum albumin, 1% polyvinylpyrrolidine, 1% Ficoll), 0.1% SDS and sheared, denatured herring testes DNA (100 µg ml$^{-1}$). cDNA probes, labelled to a specific activity of $> 5 \times 10^8$ c.p.m. µg$^{-1}$ DNA with α-$^{32}$P-dATP (3000 Ci mmol$^{-1}$; Dupont/NEN) by the random priming method (Feinberg & Vogelstein, 1983) or by nick translation (Sambrook *et al.*, 1989), were added directly to the blot in the prehybridisation solution. Prehybridisations and hybridisations were carried out at 42°C for 8–16 h. Blots were washed four times in 0.1% SDS and 0.1 × SSC (1 × = 150 mM NaCl, 15 mM sodium citrate, pH 7.0) for 15 min each at 52°C. The temperature was raised to increase stringency as required. The β-actin cDNA (201 pBV2.2) (Ueyama *et al.*, 1984) and rRNA probes were used to verify equal loading of gels. For slot blot analysis, RNA samples were denatured in 1.25 × SSC and 40% formamide for 15 min at 65°C before being applied to a prewetted (10 × SSC for 10 min) nylon membrane in a slot blot apparatus under vacuum. The RNA was fixed to the membrane by baking at 80°C *in vacuo* and the membranes probed as above. Densitometry was performed using an HSI GS-300 Scanning Densitometer (Hoefer Scientific Instruments, San Francisco, CA).

### Results

### In vitro *translation and immunoprecipitations*

The relative levels of mRNA corresponding to the 3.186 antigen were compared by immunoprecipitation of equivalent amounts of $^{35}$S-methionine labelled *in vitro* translation products of RNA from both H69 and H69AR cells. Densitometry of autoradiographs showed that 5 to 6-fold more of the 36 kDa antigen was immunoprecipitated from the translation products of H69AR mRNA relative to H69 (Figure 1). These results indicate a 5 to 6-fold difference in mRNA levels, assuming that the mRNA for the 3.186 antigen is translated with comparable efficiency in RNA preparations from both the H69 and H69AR cell lines.

### *Isolation and characterisation of 3.186 cDNA clones*

To further characterise the 3.186 mRNA and its levels of expression, cDNA clones were isolated from an H69AR cDNA library by screening with $^{125}$I-MAb 3.186. Three positive clones were obtained after secondary screening and plaque purification. All three clones contained inserts of approximately 1.4 kb with a single internal EcoRI site. Digestion with this enzyme yielded fragments of approximately 1.0 and 0.4 kb in all three cases. The presence of an internal EcoRI site was not unexpected because EcoRI adaptors (which obviate the need for EcoRI digestion of the cDNA prior to insertion in the vector) were used in the cloning procedure. The possibility that the internal EcoRI site resulted from artifactual cloning of two fragments was eliminated by the DNA sequencing results as described below. The two EcoRI fragments from the inserts of all three clones were subcloned into the phagemid vector pGEM®-3Zf(+). They were then used as probes for RNA blotting analyses and sequencing studies.

Northern blot analysis of H69 and H69AR total and poly(A$^+$) RNA showed hybridization with a single mRNA species of approximately1.4–1.6 kb (Figure 2). Densitometry showed 5 to 6-fold higher levels of this mRNA in H69AR cells relative to H69. Equal loading of RNA samples on the gel was confirmed by stripping and probing of the northern blot with a $^{32}$P-labelled β-actin cDNA probe (data not

**Figure 1** MAb 3.186 immunoprecipitations of $^{35}$S-methionine labelled *in vitro* translation products. Lanes 1 and 4, total translation products from H69 and H69AR mRNA, respectively; lanes 2 and 3 MAb 3.186 immunoprecipitations of translation products from H69 and H69AR mRNA, respectively. The position of the 36 kDa 3.186 antigen is indicated on the right and the position of the molecular weight markers (kDa) is indicated on the left.

**Figure 2** Northern blot analysis of RNAs from H69 and H69AR cells. Poly(A)$^+$ RNA (1 μg) and total RNA (30 μg) were separated by agarose gel electrophoresis and blotted to a nylon membrane. The blot was probed with the 1.0 kb EcoRI subclone of the 3.186 cDNA clone, stripped and reprobed with a β-actin cDNA. Levels of the mRNA that hybridised specifically with the 1.0 kb cDNA were estimated by densitometry and the size of the mRNA was estimated by comparison with an RNA size ladder.

shown). The relative levels of mRNA corresponding to the 3.186 antigen shown by northern blotting are in reasonable agreement with those obtained by immunoprecipitation of *in vitro* translation products (Figure 1).

Partial double stranded cDNA sequencing of the subcloned 1.0 and 0.4 kb EcoRI fragments indicated that the three clones isolated were derived from the same cDNA insert. A search of the GenBank DNA sequence database revealed a perfect match with human calpactin I (heavy chain) (accession # M14043) (Saris *et al.*, 1986) and lipocortin II (accession # A23942) (Huang *et al.*, 1986), proteins also known as annexin II and p36. Further confirmation of the identity of the 3.186 cDNA clone was obtained by restriction endonuclease mapping with AccI, NdeI, HincII, HindIII, PstI and XbaI. In all cases, digestion with these enzymes generated fragments with sizes that were in accordance with those predicted from the established sequence of p36. These data are summarised in Figure 3.

### *Comparison of 3.186 mRNA levels in H69, H69AR and H69PR cells*

Shortly after cloning the cDNAs corresponding to the 3.186 antigen, we succeeded in isolating a revertant of the H69AR cell line, designated H69PR, after very long term culture (>37 months) of H69AR cells in drug-free medium. The sensitivity of the revertant H69PR cells to DOX, vincristine and VP-16 approaches that of the sensitive H69 cells (Table I). Therefore we were able to compare 3.186 protein and mRNA levels in the H69PR cell line to those in the H69 and H69AR cell lines. Immunoblot analysis and flow cytometry showed comparable amounts of 3.186 protein in H69AR and H69PR cells (results not shown). Immunoprecipitation of *in vitro* translation products of RNA from the H69, H69AR and H69PR cell lines indicated that the levels of 3.186

**Figure 3** Summary map of the 1.4 kb cDNAs isolated from an H69AR cDNA library with MAb 3.186. The hatched bars are the 1.0 and 0.4 kb EcoRI subclones (designated MJ3 and MJ8, respectively) and the arrows indicate the portions of the cDNA that were sequenced. The restriction enzyme sites are indicated by the solid vertical lines. The stippled bar is the coding sequence of the p36 cDNA while the horizontal lines to the left and the right of the stippled bar are non-coding regions.

**Table I** Relative resistance of the H69, H69AR and H69PR cell lines

| Cell line | $IC_{50}$ μM (fold-resistant) DOX | VP-16 | Vincristine |
|---|---|---|---|
| H69 | 0.095 | 5.4 | 0.01 |
| H69AR | 6.0 (63) | >30 (>6) | 1 (100) |
| H69PR | 0.33 (3.5) | 7 (1.3) | 0.018 (1.8) |

mRNA in H69PR were the same as that seen in H69AR (Figure 4, panel a). Northern (Figure 4, panel b) and slot blot (Figure 4, panel c) analyses of the three cell lines also showed that levels of 3.186 mRNA were comparable in the resistant H69AR and revertant H69PR cell lines. The slight decrease in 3.186 mRNA in the H69PR cell line observed in the northern blot (Figure 4, panel b) is attributable to an underloading of H69PR RNA as indicated by the relative amounts of 28S rRNA.

### Discussion

The H69AR cell line is one of an increasing number of cell lines that displays multidrug resistance in the absence of elevated levels of P-glycoprotein (Mirski *et al.*, 1987; Slovak *et al.*, 1988; de Jong *et al.*, 1990; Baas *et al.*, 1990; Reeve *et al.*, 1990; Slapak *et al.*, 1990; Cass *et al.*, 1989). We have derived four MAbs that detect antigens overexpressed in H69AR cells as a means of identifying proteins that may be involved in the resistance mechanism of these cells (Mirski & Cole, 1989; Mirski & Cole, 1991). The first objective of this

**Figure 4** Analyses of relative p36 mRNA levels in the H69, H69AR and H69PR cell lines. Panel **a**, MAb 3.186 immunoprecipitation of *in vitro* translated mRNA; panel **b**, Northern blot analysis with the 1.0 kb 3.186 cDNA subclone MJ3 and 28S rRNA; panel **c**, slot blot analysis of RNA hybridised with MJ3 and 28S rRNA.

study was to determine whether the increased expression of the 3.186 antigen was a consequence of enhanced synthesis as reflected by elevated levels of its mRNA. The *in vitro* translation and immunoprecipitation experiments confirmed that this was the case (Figure 1). Our second objective was to further characterise the 3.186 mRNA and protein by isolation of 3.186 cDNA clones. The clone isolated by screening a H69AR cDNA expression library with MAb 3.186 contained an insert of approximately 1.4 kb. Fragments of the cloned DNA hybridized with a single mRNA size class also of approximately 1.4 kb (Figure 2), suggesting that the cDNA was essentially full length. DNA sequencing of three different regions of the clone revealed complete identity with the published sequence of human calpactin I (Saris *et al.*, 1986) and the GenBank database sequence of lipocortin II, proteins also referred to as p36 and annexin II. Restriction endonuclease analysis also demonstrated complete consistency with sites predicted from the DNA sequence of p36 (Figure 3). The conclusion that the 3.186 antigen is identical to p36 is further supported by the known biochemical properties of these two proteins, *viz.*, the sizes of the polypeptides are identical, both can be phosphorylated and are not detectably *N*-glycosylated (Mirski & Cole, 1991; Mel'gunov, 1991) and both can be detected in association with various cellular membranes. The 3.186 antigen has also been detected on the human colon carcinoma WiDR cell line and peripheral blood mononuclear cells (Mirski & Cole, 1991; Krebes *et al.*, 1991) as has p36 (Frohlich *et al.*, 1990; Rothhut *et al.*, 1987). Taken together, our data provide convincing evidence that the antigen reacting with MAb 3.186 is identical to p36 and that p36 synthesis is elevated in the multidrug resistant H69AR cell line.

p36 belongs to a family of calcium and phospholipid binding proteins that consists of at least eight members. These proteins are known by several names including annexins, lipocortins, calpactins, chromobindins, and calelectrins (Crumpton & Dedman, 1990; Russo-Marie, 1991). The various annexins are very similar proteins suggesting overlapping activities. However, distinct differences in some aspects of their structure and in their cellular and intracellular localisation indicate that each protein is likely to have a specialised function as well (Pepinsky *et al.*, 1988; Ernst *et al.*, 1991; Glenney *et al.*, 1987). All members of the annexin family have a core consisting of four repeated units of approximately 70 amino acids that contain phospholipid and calcium binding sites. The $NH_2$-terminal tails of the annexins are quite variable in sequence and length. In p36 (annexin II), this region contains both serine and tyrosine phosphorylation sites (Glenney & Tack, 1985; Gerke, 1989) and p36 has been shown to be a substrate for pp60$^{v\text{-}src}$ (Huang *et al.*, 1986; Erikson *et al.*, 1984; Saris *et al.*, 1986) and protein kinase C (Gould *et al.*, 1986; Barnes *et al.*, 1991).

Although much is known about the molecular and biochemical properties of p36, its true physiological functions remain uncertain (Gerke, 1989; Crompton *et al.*, 1988; Mel'gunov, 1991). p36 exists as either a monomer or as a heterotetramer with an 11 kDa protein, p11, forming a $(p36)_2(p11)_2$ complex (Erikson *et al.*, 1984; Glenney & Tack, 1985; Johnsson *et al.*, 1988; Glenney *et al.*, 1986; Zokas & Glenney, 1987). In the latter form, it may be a structural component of the cytoskeletal framework. In addition, p36 has been implicated in a number of cellular events, including signal transduction through inhibition of phospholipase $A_2$ (Davidson *et al.*, 1987; Brugge, 1986), DNA lagging strand synthesis (Jindal *et al.*, 1991) and exocytosis (Burgoyne, 1991). Finally, increased expression of p36 has been associated with transformation (Frohlich *et al.*, 1990) and differentiation (Isacke *et al.*, 1989; Tox *et al.*, 1991).

The functional significance of the overexpression of p36 with respect to drug resistance remains unknown. Previously, we examined a panel of fifteen paired drug-sensitive and -resistant tumour cell lines derived from a variety of tissues by a cell ELISA but found no resistance-associated overexpression of the 3.186 antigen (Mirski & Cole, 1991). Furthermore, comparative analyses of p36 mRNA and protein levels in H69 and H69AR cell lines with the recently obtained H69PR revertant cell line indicate that recovery of drug sensitivity is not accompanied by any grossly apparent decrease in the synthesis of p36 (Figure 4). However, the full implications of these findings are unclear. At present, we do not know whether the acquisition of resistance in H69AR cells or reversion to drug sensitivity in H69PR cells is associated with altered post-translational modifications of p36 (e.g. phosphorylation levels) and/or with altered intracellular localisation of p36. In this regard, a recent study in which the p36 protein was shown to be identical to the primer recognition protein 1 (PRP1) is of interest. PRP1 (p36) associates with the glycolytic enzyme 3-phosphoglycerate kinase (PRP2) to form a complex that interacts with DNA polymerase α (Jindal *et al.*, 1991) and thus p36 may play a major role in the coordination of leading and lagging strand DNA synthesis. It is worth noting that this important function involves less than 5% of the total cellular p36. Consequently, alterations in the amounts and/or phosphorylation of nuclear p36 could significantly affect DNA synthesis through PRP1 function without the total cellular p36 being detectably affected. Slight alterations in the subcellular distribution of p36 could similarly affect lagging strand DNA synthesis. Of further interest is the preliminary report of alterations in another annexin, annexin I (p35), in a murine DOX-selected multidrug resistant cell line (Bhushan & Tritton, 1991). In this case, the sensitive and resistant cell lines do not differ in their levels of p35 protein but in their degree of p35 phosphorylation. These investigators have postulated that phosphorylation of p35 by protein tyrosine kinases, with consequent effects on phospholipase $A_2$, may play a role in regulating the multidrug resistance phenotype. Thus some activities of p36 that may relate to drug resistance could be modulated by the level of phosphorylation and/or subcellular localisation of the protein. For this reason, comparative analyses of the phosphorylation status and intracellular distribution of p36 in the H69, H69AR and H69PR cell lines are the subject of ongoing investigations.

This work was supported by a grant from the Medical Research Council of Canada to S.P.C. Cole and R.G. Deeley. M.J. Pinkoski was supported by a studentship from the Cancer Research Society, Inc. The authors wish to thank J. Gerlach, C. Grant, K. Krebes, J. Mackie and S. Mirski for helpful advice and expert assistance and D. Clements and K. Sparks for technical support.

### References

BAAS, F., JONGSMA, A.P.M., BROXTERMAN, H.J. & 7 others (1990). Non-P-glycoprotein mediated mechanism for multidrug resistance precedes P-glycoprotein expression during *in vitro* selection for doxorubicin resistance in a human lung cancer cell line. *Cancer Res.*, **50**, 5392.

BARNES, J.A., MICHIEL, D. & HOLLENBERG, M.D. (1991). Simultaneous phosphorylation of three human calpactins by kinase C. *Biochem. Cell. Biol.*, **69**, 163.

BHUSHAN, A & TRITTON, T.R. (1991). Increased tyrosine phosphorylation of lipocortin I in multidrug resistant Sarcoma 180 cells. *Proc. AACR*, **32**, 362 (Abstract).

BRAMBILLA, E., MORO, D., GAZZERI, S. & 5 others (1991). Cytotoxic chemotherapy induces cell differentiation in small-cell lung carcinoma. *J. Clin. Oncol.*, **9**, 50.

BRUGGE, J.S. (1986). The p35/p36 substrates of protein-tyrosine kinases as inhibitors of phospholipase $A_2$. *Cell*, **46**, 149.

BURGOYNE, R.D. (1991). Control of exocytosis in adrenal chromaffin cells. *Biochim. Biophys. Acta*, **1071**, 174.

CASS, C.E., JANOWSKA-WIECZORE, A., LYNCH, M.A., SHEININ, H., HINDENBURG, A.A. & BECK, W.T. (1989). Effect of duration of exposure to verapamil on vincristine activity against multidrug-resistant human leukemic cell lines. *Cancer Res*, **49**, 5798.

COLE, S.P.C. (1986). Rapid chemosensitivity testing of human lung tumor cells using the MTT assay. *Cancer Chemother. Pharmacol.*, **17,** 259.

COLE, S.P.C. (1990). Patterns of cross-resistance in a multidrug-resistant small-cell lung carcinoma cell line. *Cancer Chemother. Pharmacol.*, **26,** 250.

COLE, S.P.C., CHANDA, E.R., DICKE, F.P., GERLACH, J.H. & MIRSKI, S.E.L. (1991). Non-P-glycoprotein-mediated multidrug resistance in a small cell lung cancer cell line: evidence for decreased susceptibility to drug-induced DNA damage and reduced levels of topoisomerase II. *Cancer Res.*, **51,** 3345.

CROMPTON, M.R., MOSS, S.E. & CRUMPTON, M.J. (1988). Diversity in the lipocortin/calpactin family. *Cell*, **55,** 1.

CRUMPTON, M.J. & DEDMAN, J.R. (1990). Protein terminology tangle. *Nature*, **345,** 212.

DAVIDSON, F.F., DENNIS, E.A., POWELL, M. & GLENNEY, J.R. Jr (1987). Inhibition of phospholipase $A_2$ by 'lipocortins' and calpactins. An effect of binding to substrate phospholipids. *J. Biol. Chem.*, **262,** 1698.

DAVIS, L.G., DIBNER, M.D. & BATTEY, J.F. (1986). *Basic Methods in Molecular Biology*, pp. 216–218. Elsevier Press: New York.

DE JONG, S., ZIJLSTRA, J.G., DE VRIES, E.G.E & MULDER, N.H. (1990). Reduced DNA topoisomerase II activity and drug-induced DNA cleavage activity in an adriamycin-resistant human small cell lung carcinoma cell line. *Cancer Res.*, **50,** 304.

DEUCHARS, K.L. & LING, V. (1989). P-glycoprotein and multidrug resistance in cancer chemotherapy. *Semin. Oncol.*, **16,** 156.

ENDICOTT, J.A. & LING, V. (1989). The biochemistry of P-glycoprotein-mediated multidrug resistance. *Annu. Rev. Biochem.*, **58,** 137.

ERIKSON, E., TOMASIEWICZ, H.G. & ERIKSON, R.L. (1984). Biochemical characterization of a 34-kilodalton normal cellular substrate of $pp60^{v\text{-}src}$ and an associated 6-kilodalton protein. *Mol. Cell. Biol.*, **4,** 77.

ERNST, J.D., HOYE, E., BLACKWOOD, R.A. & MOK, T.L. (1991). Identification of a domain that mediates vesicle aggregation reveals functional diversity of annexin repeats. *J. Biol. Chem.*, **266,** 6670.

FEINBERG, A.P. & VOGELSTEIN, B. (1983). A technique for radiolabeling DNA restriction endonuclease fragments to high specific activity. *Analyt. Biochem.*, **132,** 6.

FROHLICH, M., MOTTE, P., GALVIN, K., TAKAHASHI, H., WANDS, J. & OZTURK, M. (1990). Enhanced expression of the protein kinase substrate p36 in human hepatocellular carcinoma. *Mol. Cell. Biol.*, **10,** 3216.

GERKE, V. (1989). Tyrosine protein kinase substrate p36: a member of the annexin family of $Ca^{2+}$/phospholipid-binding proteins. *Cell Motil. Cytoskel.*, **14,** 449.

GERLACH, J.H., ENDICOTT, J.A., JURANKA, P.F. & 4 others (1986). Homology between P-glycoprotein and a bacterial haemolysin transport protein suggests a model for multidrug resistance. *Nature*, **324,** 485.

GLENNEY, J.R. Jr, BOUDREAU, M., GALYEAN, R., HUNTER, T. & TACK, B. (1986). Association of the S-100-related calpactin I light chain with the $NH_2$-terminal tail of the 36-kDa heavy chain. *J. Biol. Chem.*, **261,** 10485.

GLENNEY, J.R. Jr, TACK, B. & POWELL, M.A. (1987). Calpactins: two distinct $Ca^{++}$-regulated phospholipid- and actin-binding proteins isolated from lung and placenta. *J. Cell Biol.*, **104,** 503.

GLENNEY, J.R. Jr & TACK, B.F. (1985). Amino-terminal sequence of p36 and associated p10: identification of the site of tyrosine phosphorylation and homology with S-100. *Proc. Natl Acad. Sci. USA*, **82,** 7884.

GOULD, K.L., WOODGETT, J.R., ISACKE, C.M. & HUNTER, T. (1986). The protein-tyrosine kinase substrate p36 is also a substrate for protein kinase C *in vitro* and *in vivo*. *Mol. Cell Biol.*, **6,** 2738.

HUANG, K.-S., WALLNER, B.P., MATTALIANO, R.J. & 12 others (1986). Two human 35 kd inhibitors of phospholipase $A_2$ are related to substrates of $pp60^{v\text{-}src}$ and of the epidermal growth factor receptor/kinase. *Cell*, **46,** 191.

ISACKE, C.M., LINDBERG, R.A. & HUNTER, T. (1989). Synthesis of p36 and p35 is increased when U-937 cells differentiate in culture but expression is not inducible by glucocorticoids. *Mol. Cell. Biol.*, **9,** 232.

JINDAL, H.K., CHANEY, W.G., ANDERSON, C.W., DAVIS, R.G. & VISHWANATHA, J.K. (1991). The protein-tyrosine kinase substrate, calpactin I heavy chain (p36), is part of the primer recognition protein complex that interacts with DNA polymerase α. *J. Biol. Chem.*, **266,** 5169.

JOHNSSON, N., MARRIOTT, G. & WEBER, K. (1988). p36, the major cytoplasmic substrate of src tyrosine protein kinase, binds to its p11 regulatory subunit via a short amino-terminal amphiphatic helix. *EMBO J.*, **7,** 2435.

KREBES, K.A., MIRSKI, S.E.L., PROSS, H.F. & COLE, S.P.C. (1991). Expression of multidrug resistance-associated antigens on peripheral blood mononuclear cells. *Proc. Can. Fed. Biol. Soc.*, **34,** 8 (Abstract).

LAI, S.-L., GOLDSTEIN, L.J., GOTTESMAN, M.M. & 7 others (1989). MDR1 gene expression in lung cancer. *J. Natl Cancer Inst.*, **81,** 1144–1150.

MARKWELL, M.A.K. (1982). A new solid-state reagent to iodinate proteins. *Analyt. Biochem.*, **125,** 427.

MEL'GUNOV, V.I. (1991). Annexins – a new family of $Ca^{2+}$-binding proteins. *Biochemistry-USSR*, **56,** 107.

MIRSKI, S.E.L., GERLACH, J.H. & COLE, S.P.C. (1987). Multidrug resistance in a human small cell lung cancer cell line selected in adriamycin. *Cancer Res.*, **47,** 2594.

MIRSKI, S.E.L. & COLE, S.P.C. (1989). Antigens associated with multidrug resistance in H69AR, a small cell lung cancer cell line. *Cancer Res.*, **49,** 5719.

MIRSKI, S.E.L. & COLE, S.P.C (1991). Multidrug resistance-associated antigens on drug-sensitive and -resistant human tumour cell lines. *Br. J. Cancer*, **64,** 15.

NOONAN, K.E., BECK, C., HOLZMAYER, T.A. & 8 others (1990). Quantitative analysis of *MDR1* (multidrug resistance) gene expression in human tumors by polymerase chain reaction. *Proc. Natl Acad. Sci. USA*, **87,** 7160.

PEPINSKY, R.B., TIZARD, R., MATTALIANO, R.J. & 11 others (1988). Five distinct calcium and phospholipid binding proteins share homology with lipocortin I. *J. Biol. Chem.*, **263,** 10799.

REEVE, J.G., RABBITS, P.H. & TWENTYMAN, P.R. (1990). Non-P-glycoprotein-mediated multidrug resistance with reduced EGF receptor expression in a human large cell lung cancer cell line. *Br. J. Cancer*, **61,** 851.

RIORDAN, J.R., DEUCHARS, K., KARTNER, N., ALON, N., TRENT, J. & LING, V. (1985). Amplification of P-glycoprotein genes in multidrug-resistant mammalian cell lines. *Nature*, **316,** 817.

ROTHHUT, B., COMERA, C., PRIEUR, B., ERRASFA, M., MINASSIAN, G. & RUSSO-MARIE, F. (1987). Purification and characterization of a 32-kDa phospholipase $A_2$ inhibitory protein (lipocortin) from human peripheral blood mononuclear cells. *FEBS Lett.*, **219,** 169.

RUSSO-MARIE, F. (1991). Lipocortins: an update. *Prostaglan. Leuk. Ess. Fatty Acids*, **42,** 83.

SAMBROOK, J., FRITSCH, E.F. & MANIATIS, T. (1989). *Molecular Cloning: A Laboratory Manual.* Cold Spring Harbor Laboratory Press: Cold Spring Harbor, NY.

SANGER, F., NICKLEN, S. & COULSON, A.R. (1977). DNA sequencing with chain-terminating inhibitors. *Proc. Natl Acad. Sci. USA*, **74,** 5463.

SARIS, C.J.M., TACK, B.F., KRISTENSEN, T., GLENNEY, J.R. Jr & HUNTER, T. (1986). The cDNA sequence for the protein-tyrosine kinase substrate p36 (calpactin I heavy chain) reveals a multidomain protein with internal repeats. *Cell*, **46,** 201.

SLAPAK, C.A., DANIEL, J.C. & LEVY, S.B. (1990). Sequential emergence of distinct resistance phenotypes in murine erythroleukemia cells under adriamycin selection: decreased anthracycline uptake precedes increased P-glycoprotein expression. *Cancer Res.*, **50,** 7895.

SLOVAK, M.L., HOETGE, G.A., DALTON, W.S. & TRENT, J.M. (1988). Pharmacological and biological evidence for differing mechanisms of doxorubicin resistance in two human tumor cell lines. *Cancer Res.*, **48,** 2793.

TOX, M.T., PRENTICE, D.A. & HUGHES, J.P. (1991). Increases in p11 and annexin II proteins correlate with differentiation in the PC12 pheochromocytoma. *Biochem. Biophys. Res. Commun.*, **177,** 1188.

UEYAMA, H., HAMADA, H., BATTULA, N. & KAKUNAGA, T. (1984). Structure of a human smooth muscle actin gene (aortic type) with a unique intron site. *Mol. Cell. Biol.*, **4,** 1073.

ZOKAS, L. & GLENNEY, J.R. Jr (1987). The calpactin light chain is tightly linked to the cytoskeletal form of calpactin I: studies using monoclonal antibodies to calpactin subunits. *J. Cell. Biol.*, **105,** 2111.